# The Transplanting and Replanting of Teeth

**Published:** 1884-12

**Authors:** A. H. Fuller

**Affiliations:** St. Louis


### THE TRANSPLANTING AND REPLANTING OF TEETH.

BY A. H. FULLER, M. D., ST. LOUIS.

Dr. Wm. N. Morrison, in a paper under the above heading, read before the St. Louis Dental Society, and published in the July number of the *Missouri Dental Journal*, 1882, says, page 244: "Among all such cases produced by others I have never seen a living pulp, which is my reason for attributing to the dead pulp the whole responsibility for the trouble. In 1862 I performed that operation with that distinctive feature for the first time, reports of which will be found in the transactions of the American Dental Association for 1875 and 1876, wherein I described what I pronounce the indispensable operation of the removal of the root vessels and a thorough filling of the root canals. I can find no record of this method of operation being performed prior to 1862."

In "The History and Treatment of the Diseases of the Teeth, etc.," by Joseph Fox, 1806, page 56, under a chapter on "Fractures of the Teeth," he says: "In plate III., figure 9, is a representation of two central incisors which were broken by a fall. Figure 10 is the posterior view of these teeth, the fracture of which will be seen extending into the cavity.

"In an accident of this kind, affecting either one or both teeth, if the person should apply for assistance immediately after the accident, and before any inflammation has supervened, I should recommend that the tooth or teeth be extracted with great care.

"When this has been done, the cavity in the tooth should be cleared out as much as psssible, and some gold leaf be introduced, so as completely to fill it up.

"After the cavity has been thus stopped, the teeth are to be restored to their sockets, and there to be confined by a ligature; they will soon fix, and in a few days be as secure as ever, and may afterwards remain for a great number of years without inconvenience."

The attention of the dental profession is called to this subject, simply as a matter of history—Mr. Fox recommending the operation nearly eighty years ago, and forty years before filling the pulp canals in teeth was generally practiced.

